# Single-cell analysis of immune cell transcriptome during HIV-1 infection and therapy

**DOI:** 10.1186/s12865-022-00523-2

**Published:** 2022-09-29

**Authors:** Justin Pollara, Santosh Khanal, R. Whitney Edwards, Bhavna Hora, Guido Ferrari, Barton F. Haynes, Todd Bradley

**Affiliations:** 1 Department of Surgery, Duke University Medical Center, Durham, NC 27710 USA; 2 Duke Human Vaccine Institute, Duke University Medical Center, Durham, NC 27710 USA; 3 Genomic Medicine Center, Children’s Mercy Kansas City, Kansas City, MO 64108 USA; 4 Department of Medicine, Duke University Medical Center, Durham, NC 27710 USA; 5 Department of Immunology, Duke University Medical Center, Durham, NC 27710 USA; 6 Department of Pediatrics, University of Missouri at Kansas City School of Medicine, Kansas City, MO 64108 USA; 7 Department of Pediatrics, University of Kansas Medical Center, Kansas City, KS 66160 USA

**Keywords:** HIV-1, Single-cell RNA-seq, HIV-1 infection, Immune cells

## Abstract

**Background:**

Cellular immune responses are phenotypically and functionally perturbed during HIV-1 infection, with the majority of function restored upon antiretroviral therapy (ART). Despite ART, residual inflammation remains that can lead to HIV-related co-morbidities and mortality, indicating that ART does not fully restore normal immune cell function. Thus, understanding the dynamics of the immune cell landscape during HIV-1 infection and ART is critical to defining cellular dysfunction that occurs during HIV-1 infection and imprints during therapy.

**Results:**

Here, we have applied single-cell transcriptome sequencing of peripheral blood immune cells from chronic untreated HIV-1 individuals, HIV-1-infected individuals receiving ART and HIV-1 negative individuals. We also applied single-cell transcriptome sequencing to a primary cell model of early HIV-1 infection using CD4+ T cells from healthy donors. We described changes in the transcriptome at high resolution that occurred during HIV-1 infection, and perturbations that remained during ART. We also determined transcriptional differences among T cells expressing HIV-1 transcripts that identified key regulators of HIV-1 infection that may serve as targets for future therapies to block HIV-1 infection.

**Conclusions:**

This work identified key molecular pathways that are altered in immune cells during chronic HIV-1 infection that could remain despite therapy. We also identified key genes that are upregulated during early HIV-1 infection that provide insights on the mechanism of HIV-1 infection and could be targets for future therapy.

**Supplementary Information:**

The online version contains supplementary material available at 10.1186/s12865-022-00523-2.

## Background

HIV-1 infection leads to progressive loss of CD4+ T cells that results in the development of acquired immunodeficiency syndrome (AIDS) [1, 2]. Initiation and adherent use of antiretroviral therapy (ART) can achieve viral suppression that decreases HIV-1 mortality and reduces the potential to transmit the virus to others [3–5]. In addition, the chronic inflammation caused by the virus has been linked to non-AIDS related morbidity and mortality making it clear that HIV-1 damages the host immune system and ART may only partially restore the damage and the level of activation of the immune system [6–8]. These observations coupled with the number of new HIV-1 infections that occur despite prevention and therapeutic efforts highlight the need to better understand the impact HIV-1 has on host immune cell function in order to understand infection and improve therapeutic outcomes.

Recently, high-throughput single-cell RNA sequencing (scRNA-seq) has emerged as a powerful tool to understand the transcriptional differences in populations of cells at single-cell resolution and has provided critical insights to understanding human disease pathogenesis. Single-cell approaches have been used to study HIV-1 replication and infection, the heterogeneity of the HIV-1 latent reservoir and the cellular response to latency reversal agents [9–13]. Many of these studies have focused on the effect of HIV-1 on CD4+ T cells and have identified key pathways that are important for the regulation of HIV-1 infection and latency. Study of non-CD4+ T cell populations has been limited, but investigators have demonstrated the complex interplay of innate and adaptive immunity in the regulation of the HIV-1 broadly neutralizing antibody responses and viremia during infection [14–16].

Here, we sequenced the transcriptomes of 81,235 peripheral blood immune cells from six untreated chronic HIV-1-infected individuals, three ART-treated HIV-1-infected individuals and three HIV-1 seronegative individuals. We also identified cell types and states that changed during infection and described changes in immune cells that remain during ART. Finally, we used the scRNA-seq approach in a primary cell model of acute infection with HIV-1 to identify gene expression changes that occur in cells that express HIV-1 compared to those without HIV-1 transcript expression.

## Results

### scRNA-seq identified distinct immune cell populations during HIV-1 infection

**Fig. 1:**
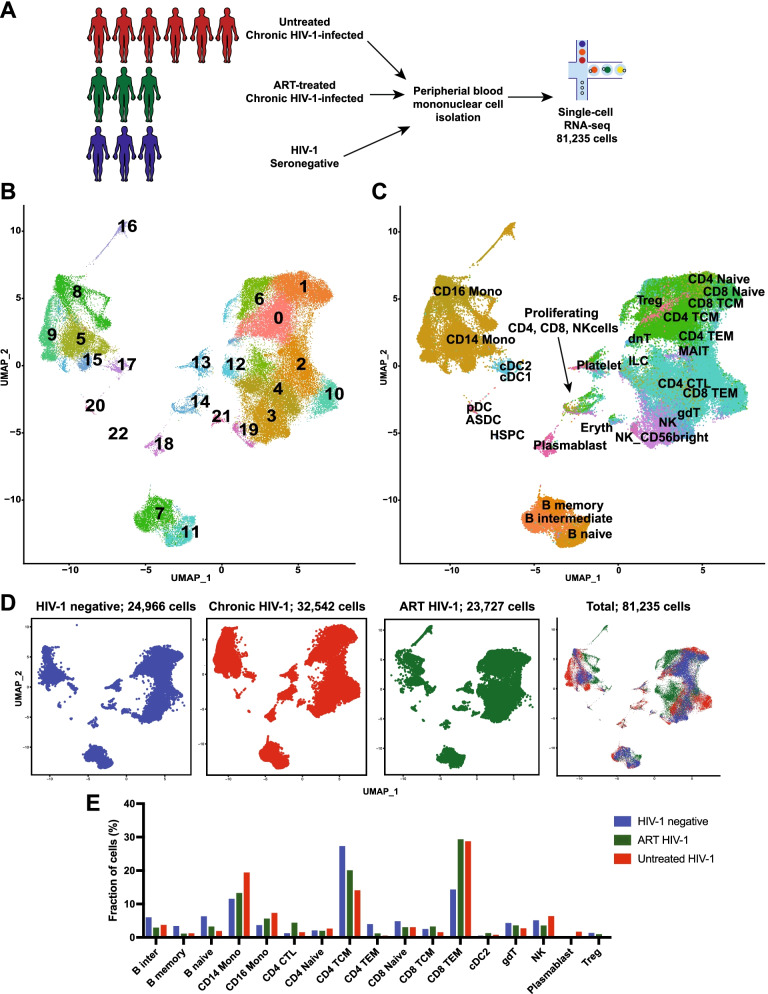
scRNA-seq of peripheral immune cells identified distinct cellular populations. **A** Schematic of scRNA-seq experimental procedure using peripheral blood mononuclear cells (PBMC) from 6 untreated chronic HIV-1-infected individuals, 3 ART-treated chronic HIV-1-infected individuals and 3 HIV-1 seronegative individuals resulting in the analysis of 81,235 total PBMC cells. **B** UMAP analysis of all 81,235 cells and graph-based clustering of cell populations resulted in the identification of 23 transcriptionally distinct clusters. Individual cells are colored by cluster identity. **C** UMAP analysis with cell colored and labeled by cell identify determined by Azimuth reference. **D** UMAP analysis with individual cells colored by sample group. **E** Bar graph showing the fraction of total single cells in each Azimuth defined cell type by group

To determine the host immune cell transcriptional changes during HIV-1 infection and ART, we performed scRNA-seq of peripheral blood mononuclear cells (PBMCs) from six untreated chronically HIV-1-infected (untreated), three ART-treated HIV-1-infected (treated) and three HIV-1 seronegative control (seronegative) participants using the 10 × Genomics scRNA-seq platform (Table 1). The HIV-1-infected individuals on ART had undetectable viral load, but were only on ART for less than one year which could allow for residual viral replication compared to individuals on ART for longer periods. In order to normalize the data and to filter out low-quality cells, we utilized the CellRanger aggregation pipeline which normalized for effective sequencing depth of each sample by subsampling the reads to the sample with the lowest depth. Next, we excluded cells that had greater than 20% of the genes that were mitochondrial genes (Additional file 1: Fig. S1A). Finally, to correct for any batch effects we used the Seurat analysis pipeline Multi CCA method to regress out cell–cell variation in gene expression in order to control for technical variation. This resulted in the elimination of 994 cells from the final dataset. Among all individuals, we determined the transcriptomes of 81,297 single cells detecting a median of 951 genes per cell (Fig. 1A; Additional file 1: Table S1).

**Table 1:** Individual demographic and clinical information

Sample ID	Sample group	Gender	Race/ethnicity	Age	Viral load	CD4 count	ART
0036	HIV-1 seronegative	Male	Ngoni	45	NA	713	NA
1363	HIV-1 seronegative	Male	Chewa	34	NA	615	NA
1628	HIV-1 seronegative	Male	Lomwe	26	NA	908	NA
0501	HIV-1 untreated	Male	Black/African American	37	15,651	514	NA
0010	HIV-1 untreated	Male	Tumbuka	25	436,975	317	NA
0468	HIV-1 untreated	Male	Chewa	27	101,634	300	NA
0694	HIV-1 untreated	Female	Lomwe	26	> 750,000	301	NA
0782	HIV-1 untreated	Male	Xhosa	30	18,169	389	NA
0391	HIV-1 untreated	Female	Xhosa	26	> 750,000	236	NA
0592	HIV-1 ART treated	Male	Black/African American	24	< 400	566	Atripla (243 days)
0010	HIV-1 ART treated	Male	Tumbuka	25	< 400	243	Triomune (243 days)
0694	HIV-1 ART treated	Female	Lomwe	26	< 400	493	Triomune (191 days)

We first used an unsupervised analysis approach and performed dimensionality reduction using Uniform Manifold Approximation and Projection (UMAP) and graph-based clustering to visualize and identify 23 transcriptionally distinct cell clusters in the total single cell dataset (Fig. 1B). To define broad cell types contained in each cluster we used the SingleR reference-based mapping to assign cell type identities to each cluster using both the Immune cell expression and Monaco Immune databases (Additional file 1: Table S2). In parallel, we determined the pattern of expression of transcripts that define immune cell identity to further classify the cell clusters. We determined the expression of *CD14* expressed in monocytes; *CD3D*, *CD4*, *CD8A* expressed in T cell populations; *KLRC1* expressed in natural killer (NK) cell populations; and *CD79A* expressed in B cell populations (Additional file 1: Fig. S2A). We then identified the genes that were uniquely upregulated in each cell cluster, and used the top three upregulated genes in each cluster to generate a heatmap of gene expression across the clusters (Fig. 1D; Additional file 2: Table S3).

Next, we used a supervised analysis approach to identify cell types but also more specific cell states using the Azimuth reference-based mapping pipeline using refence PBMC datasets. This identified naïve, memory, cytotoxic and regulatory populations of both CD4 and CD8 T cells, natural killer (NK) cell, B cells, monocyte, dendritic cells and small populations or platelet and erythroid cells (Fig. 1C). This analysis recapitulated many of the expected immune cell lineages present in PBMCs.

We identified cells by the HIV group and found that cells from HIV-1 infection (treated and untreated) had overlapping populations with the HIV-1 seronegative samples indicating overall similar transcriptomes of certain cellular populations, as well as clusters of cells that displayed a unique transcriptome (Fig. 1D; Additional file 1: Fig. S2). We then determined the fraction of cells in each Azimuth-identified cell type that was had a frequency above 1% of the total cells (Fig. 1E). The most abundant cell types in all groups were CD4 T central memory, CD8 T effector memory and CD14 monocytes. Individuals with HIV-1 infection (treated and untreated) had increased frequency of CD8 T effector memory cells (29.2%, ART HIV-1; 28.6%, Untreated HIV-1) compared to seronegative individuals (14.2%; Fig. 1E). Conversely, HIV-infected individuals had reduced frequencies of CD4 T central memory cells (20.0%, ART HIV-1; 14.0%, Untreated HIV-1) compared to seronegative individuals (27.2%; Fig. 1E). Individuals with treated or untreated HIV-1 infection also had increased frequencies of CD14 (13.2% ART HIV-1; 19.3%, Untreated HIV-1) and CD16 (5.5%, ART HIV-1; 7.2%, Untreated HIV-1) monocyte populations compared to the CD14 (11.5%) and CD16 (3.6%) monocyte populations in seronegative individuals (Fig. 1E). The reduction in CD4 T cell and increase in CD8 T and monocyte populations were also evident when the fraction of cells within each unsupervised defined cluster (Additional file 1: Fig. S2C). Despite ART treatment and no detectable HIV-1 viral load, transcriptionally unique populations of immune cells were still present in the treated group especially among monocytes and T cells (clusters 4, 6, 12, 16), indicating that ART did not fully restore the immune cell transcriptional landscape after infection (Figs. 1E; Additional file 1: Fig. S2). Cluster 6 represented over 20% of the cells in the treated group and contained a population of regulatory T cells. Clusters 12 and 16 were also nearly exclusively made up of cells in the treated group and represented populations of innate like cells (ILC) and CD16 monocytes, respectively (Additional file 1: Fig. S2C; Additional file 2: Table S3). These data revealed that HIV infection perturbed immune cell identity and that short-term treatment to undetectable HIV-1 viral load did not fully restore the cellular phenotypes.

### Identification of differentially regulated genes in immune cells from HIV-1-infected and seronegative individuals

**Fig. 2:**
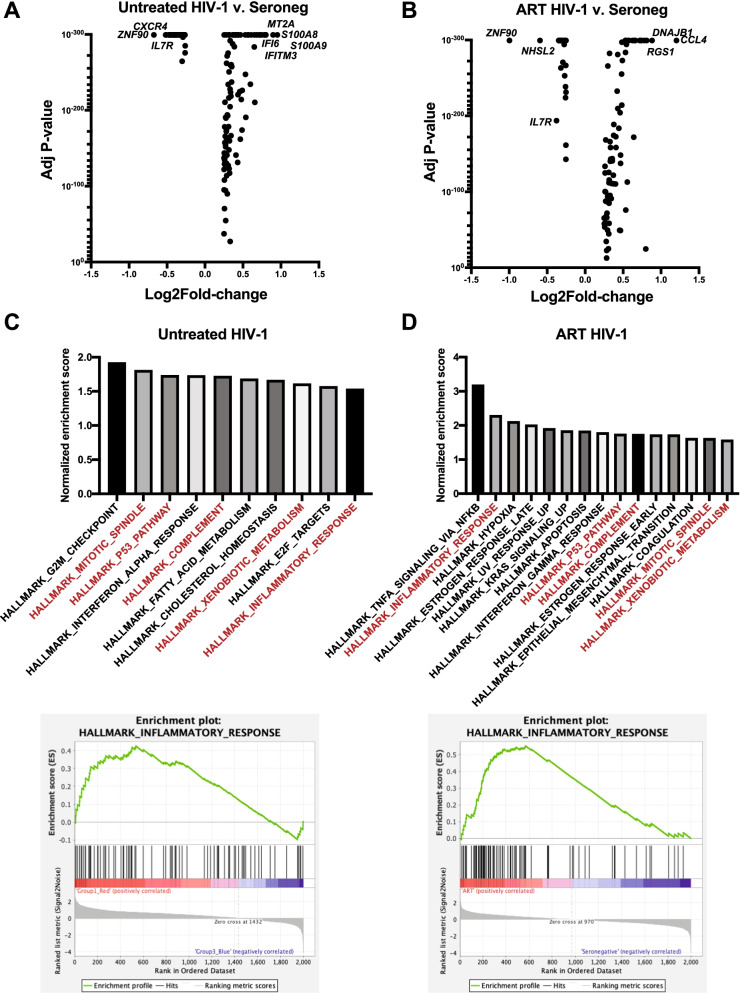
Differentially expressed genes in untreated chronic and ART treated HIV-1-infected compared with HIV-1 seronegative cells. **A**–**B** Volcano plot of genes that are significantly (P ≤ 0.05; Wilcoxon rank sum test, Bonferroni adjusted) different when comparing (A) Untreated HIV-1 vs. Seronegative and **B** ART HIV-1 vs. Seronegative group cells. X-axis displays log_2_ fold-change of each significant gene. **C**–**D** Gene Set Enrichment Analysis (GSEA) of the top enriched biological pathways in genes significantly altered in **C** untreated HIV-1 or **D** DART HIV-1. Pathways highlighted in red are present in both groups. GSEA enrichment plot for the Hallmark_Inflammatory_Response feature is shown below each graph

We next determined the genes that were differentially expressed in cells from the untreated HIV-1-infected individuals compared with the seronegative individuals. We found 96 genes that were upregulated and 45 genes that were downregulated in the cells from untreated HIV-1-infected individuals (Fig. 2A; Additional file 3: Table S4). The top seven upregulated genes are all associated with interferon response (*S100A8/9*, *MT2A*, *IFITM3*, *IFI6*, *ISG15*) [17], while the top downregulated gene *ZNF90* is a transcription factor with unknown roles in immune cells followed by the genes *CXCR4*, *IL7R* and *BTG1*, which are key regulators of lymphocyte proliferation and motility.

Next, we identified transcripts that were differentially expressed in cells from HIV-1-treated individuals compared with the HIV-1 seronegative individuals to determine genes that are changed during HIV-1 infection despite ART control of viral load. We found 83 genes upregulated and 31 genes downregulated in cells from the HIV-1-treated individuals compared to seronegative individuals (Fig. 2B; Additional file 4: Table S5). *CCL4* was the highest upregulated transcript in cells from treated individuals. The *CCL4* gene encodes the protein MIP-1b that can bind HIV-1 co-receptor CCR5 and inhibit infection [18]. The next three upregulated genes in treated individuals were *DNAJB1*, which is a part of the HSP40 complex that has been shown to play a role in anti-inflammatory processes in autoimmunity [19]; and *RGS1* and *SOD1*, which are important for antiviral immune responses [20]. The top downregulated transcripts in cells from treated individuals were the transcription factor *ZNF90*; *NHSL2*, which has unknown function; and *IL7R*, which has been shown to play a role in CD4 T cell loss during HIV-1 infection [21].

We performed Gene Set Enrichment Analysis (GSEA) using the differentially expressed transcripts in untreated HIV-1 or ART-treated HIV-1 compared to seronegative individuals and plotted the hallmark pathways that were significantly enriched (Fig. 2C, D). There were 10 hallmarks that had significant FDR-corrected q-values for the untreated HIV-1 compared to seronegative controls and 15 hallmarks that were significant in the ART-treated individuals (Additional file 1: Table S6 and S7). Both gene sets were enriched for pathways involved in inflammatory responses, complement immune responses and interferon responses (Fig. 2C, D). There were also several enriched pathways involved in metabolism and cell death. The Hallmark Inflammatory Response was significantly enriched in both HIV-1-infected groups (Fig. 2C, D). These data demonstrated that genes associated with the interferon response are upregulated in cells during HIV-1-infection, and key immune genes important for inflammatory processes and controlling virus replication, such as *CCL4*, remain changed even during ART therapy.

### T cells from ART-treated individuals have increased regulatory transcripts

**Fig. 3:**
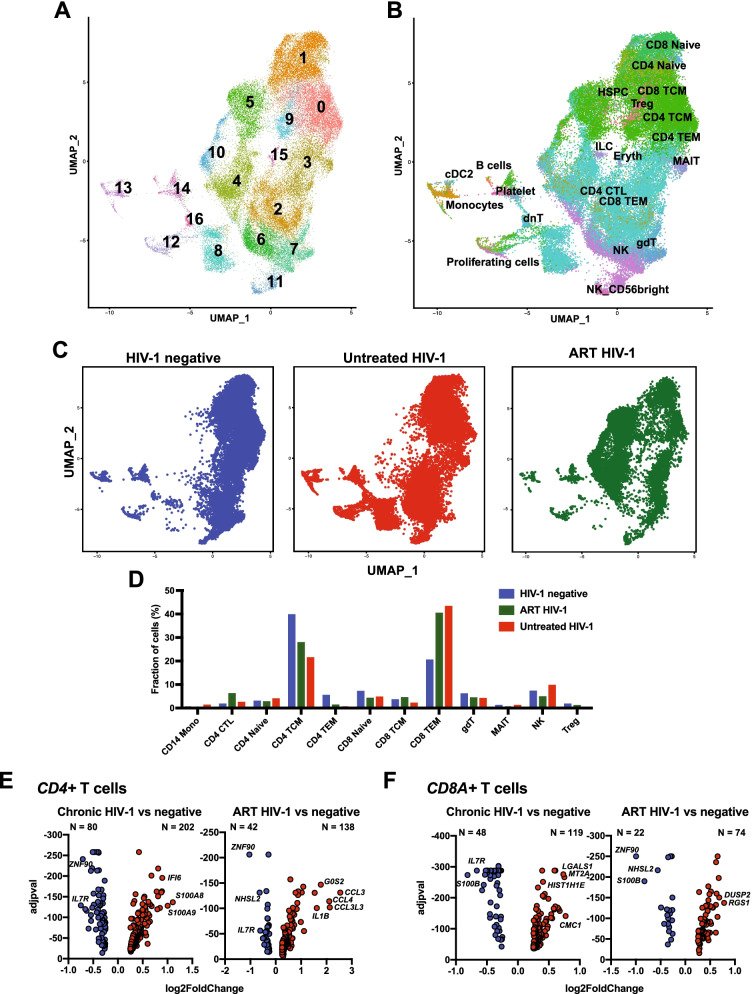
Proinflammatory signatures in T cells despite therapy. **A** UMAP plot identifying T cell clusters that were isolated and reclustered for more high-definition analysis revealing multiple cellular clusters. **B** UMAP analysis with cell colored and labeled by cell identify determined by Azimuth reference. **C** UMAP plots of T cell clusters with cells labeled and colored by experimental group. **D** Bar graph showing the fraction of total single cells in each Azimuth defined cell type by group. **E**–**F** Differentially expressed genes in Chronic or ART-treated HIV-1-infection compared to cells from seronegative individuals in **E** CD4 T cells or **F** CD8 T cells. Each dot represents a gene and Bonferroni adjusted p value displayed on Y-axis and the Log2 Fold change of expression displayed on the X-axis

We selected and performed graph-based clustering of cells that identified as T cells and NK cells from the larger dataset to determine higher resolution differences in cellular clusters (Clusters 0, 1, 2, 3, 4, 6, 10, 12, 13, 14, 19, 21; Fig. 1). We identified 17 new distinct transcriptional clusters within this subset of cells (Fig. 3A). We next determined the genes that were upregulated within each cluster of cells, and generated a heatmap of the top five genes that defined each cluster to further classify the identity of each cell cluster (Additional file 1: Fig. S3A; Additional file 5: Table S8). We also determined the expression of nine specific lineage-defining transcripts in order to further classify the cell clusters (Additional file 1: Fig. S3B). *CD3D* encodes for CD3 which identified T cell populations and the T cells were further subdivided into CD4+ and CD8+ T cells using *CD4* and *CD8A* expression, respectively. This analysis identified a single major cluster of CD4 T cells, but multiple CD8 T cell clusters that were transcriptionally distinct (Additional file 1: Fig. S3B). We found that the T cells were segregated by naïve T cell marker *CCR7* as well as a marker of T cell activation *MKI67*. There were also smaller populations of T cells with the regulatory marker *FOXP3* and exhaustion marker PD-1 (*PDCD1*; Additional file 1: Fig. S3B). We also identified two major clusters of cells that lacked T cell markers (cluster 11 and 13). Cluster 11 was further classified as NK cells due to expression of granzyme B and CD16 transcripts (*GZMB* and *FCER1G*) and cluster 13 had markers of monocyte populations (CD16, *FCER1G*; lysozyme, *LYZ*; Additional file 1: Fig. S3). In parallel, we utilized the reference-based cell identify mapping approach Azimuth to identify cell populations. This revealed similar cellular populations of CD4 and CD8 naïve and memory populations, T regulatory cells, and NK cells on the major group of single-cells on the UMAP. There were also smaller segregated populations of proliferating cell types, and platelets, B cells, monocytes and dendritic cells (Fig. 3B).

Cells from untreated HIV-1 and seronegative individuals had many overlapping clusters, while the cells from the ART-treated individuals had more unique clusters of T cells (Fig. 3C). Similar to what was observed when analyzing the entire PBMC dataset, there were smaller frequencies of CD4 TCM and increased frequencies of CD8 TEM cell populations in the cells from the untreated or treated HIV-1 infected individuals compared to the cells from the seronegative individuals (Fig. 3D). There was a shift in the T cell populations for ART-treated HIV-1 individuals that were made up of clusters 4, 5 and 10 and represented mostly CD4 cytotoxic T lymphocytes (CTL; Fig. 3A–D). These cells represented 6.14% of the NK/T cells from ART-treated individuals compared to 2.46% and 1.67% of the untreated HIV-1 and seronegative groups, respectively (Fig. 3D). Notably, these clusters had upregulation of two genes that have been shown to play a role in the development of regulatory T cells *NFKBIA* and *FTH1* [22] (Additional file 1: Fig. S3A).

Next we performed differential gene expression comparisons of the CD4+ and CD8+ T cell populations. CD4+ and CD8+ T cells (*CD4* and *CD8A*-expressing cells, respectively) from untreated HIV-1 individuals had upregulation of interferon response genes (*IFI6*, *MT2A*, *S100A8*, *S100A9*) and downregulation of the transcription factor *ZNF90* and *IL7R* that encodes CD127, important for T cell differentiation [23], when compared to cells from seronegative donors (Additional file 6: Table S9 and Additional file 7: Table S10). When comparing differentially expressed genes in cells from ART individuals and seronegative, we found that CD4+ T cells had upregulation of chemokines and *IL1B*, which contribute to the proinflammatory state (Fig. 3E; Additional file 8: Table S11). In CD8+ T cells from untreated HIV-1-infected compared to seronegative individuals, there is upregulation of *LGALS1* that encodes galectin-1 that has been shown to induce apoptosis in effector T cells and increase immunoregulatory roles of T cells [24] (Fig. 3F; Additional file 9: Table S12). CD8+ T cells had upregulation of *DUSP2* and *RGS1* that are important for T cell inflammation and cell migration [25] (Fig. 3F; Additional file 9: Table S12). We observed a similar downregulation of *ZNF90* and *IL7R* when we compared ART to seronegative cells as when we compared untreated HIV cells were compared to seronegative, indicating these transcripts are changed in T cells during untreated or treated HIV-1 infection (Fig. 3E, F). These data demonstrated that CD4+ and CD8+ T cells remain in a proinflammatory state despite ART.

### Increased expression of T cell activation and long-noncoding RNA transcripts regulate HIV-1 infection

**Fig. 4:**
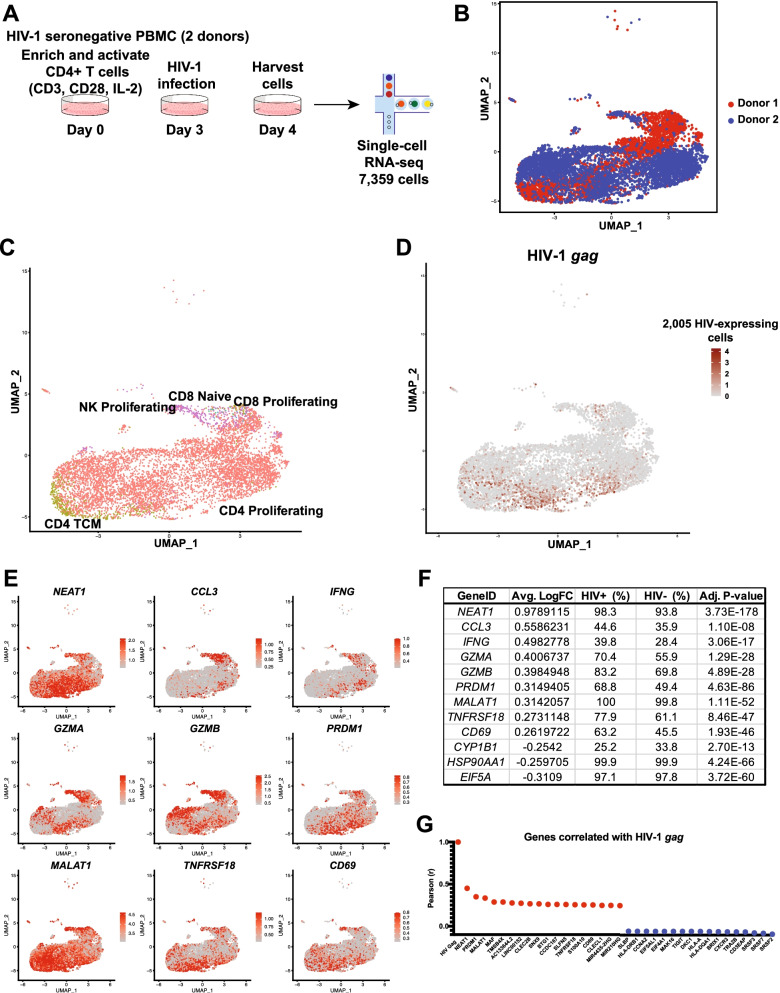
CD4 T cell activation status and long-noncoding RNAs regulate early HIV-1 infection **A** overview of primary cell model of HIV-1 infection and scRNA-seq. **B** UMAP plot of scRNA-seq of T cell data from two independent healthy individuals. Each point represents a single cell. **C** UMAP analysis with cell colored and labeled by cell identify determined by Azimuth reference. **D** UMAP plot displaying HIV-1 *gag* gene expression in the individual cells. **E** UMAP plots showing expression of transcripts that were significantly upregulated in T cells that had expression of HIV-1 *gag* compared to cells that lacked expression. **F** Transcripts that were significantly changed in T cells that had expression of HIV-1 *gag* compared to cells that laced detectable expression. **G** Top genes that correlated and anti-correlated (r, Pearson) with HIV-1 *gag* expression in all cells

To identify genes that are critical for regulating CD4+ T cell resistance or sensitivity to initial HIV-1 infection, we used scRNA-seq in a primary CD4+ T cell model of HIV-1 infection. CD4+ T cells were enriched from the PBMCs of two healthy HIV-1 uninfected donors and stimulated for 72 h with recombinant human IL-2 and antibodies directed against CD3 and CD28. After stimulation, the cells were infected with a full-length primary HIV-1 infectious molecular clone (CH058; [26]) for 24 h. Cells were harvested and subjected to scRNA-seq (Fig. 4A). We analyzed 7359 single T cells from the two donors after filtering for poor quality cells (Additional file 1: Fig. S1B). The cells from both donors clustered similarly using UMAP (Fig. 4B). Annotation of reference-based cell identities using Azimuth revealed that most cells were CD4 proliferating cells, with small populations of cells with CD4 TCM identity (Fig. 4C). There were also some cells that had gene expression profiles that matched CD8 naïve, CD8 and NK proliferating cells (Fig. 4C). Expression of HIV-1 *gag* transcripts was detected in 2005 of the cells, indicating that after 24 h of virus exposure approximately 27% of the cells had active viral transcription (Fig. 4D). In addition to HIV-1 *gag* transcript, we identified 12 genes differentially expressed in cells with HIV-1 transcripts compared with HIV-1 negative cells (Fig. 4E, F). Two long-noncoding RNAs (*NEAT1* and *MALAT1*) were upregulated as were other markers of T cell activation and interferon signaling (*CD69*, *CCL3*, *TNFRSF18*, *GZMB*, *GZMA*, *PRDM1*, *IFNG*; Fig. 4E, F). The three downregulated transcripts had variable function in cell metabolism, transcription and immune signaling (*HSP90AA1*, *EIF5A*, *CYP1B1*; Fig. 4F). Notably, the top three genes that correlated (Pearson) with HIV-1 *gag* expression levels were the two long-noncoding RNAs *NEAT1* and *MALAT1*, and *PRDM1*, which encodes the transcriptional regulator BLIMP1 (Fig. 4G; Additional file 10: Table S13). All three of these genes have been implicated in controlling HIV-1 virus transcription and latency [27–29]. Among the genes that anti-correlated with *gag* expression were members of the SR protein splicing factor family (*SRSF2*, *SRSF3*, *SRSF7*) that have been shown to be important for HIV-1 RNA splicing [30] (Fig. 4G; Additional file 10: Table S13). These data show that the two long-noncoding RNAs *NEAT1* and *MALAT1*, SR protein splicing factors, and T cell activation status are critical for acute HIV-1 infection and viral gene transcription. Future studies will be required to determine if manipulation of these genes affects sensitivity to HIV-1 infection will be required.

## Discussion

In this study, we have applied single-cell transcriptome sequencing to map at high resolution the alterations and cellular dynamics associated with HIV-1 infection and therapy. Chronic infection with HIV-1 causes progressive loss of immune cell function that leads to impaired immunity [31]. This immune dysfunction is partially restored through antiretroviral therapy, but the molecular mechanisms of the development and restoration of immune cell exhaustion are not fully understood, nor the immune cell perturbations that remain despite therapy [32, 33]. Immune activation during HIV-1 infection is not limited to the target CD4+ T cells but engages a range of molecular and cellular processes in both the innate and adaptive arms of the immune system [34]. Using single-cell transcriptomics, we were able to identify immune cell perturbations in subsets of immune cells that were changed in individuals with HIV-1 and some of which persisted in individuals on therapy.

Confirming previously published studies, HIV-1 infection exhibited a proinflammatory immune state in all immune cell types we analyzed, with evidence of upregulation of interferon response genes [13, 35, 36]. When comparing immune cells from ART-treated HIV-1-infected individuals, we found higher frequencies of activated B cells and proinflammatory CD4+ and CD8+ T cell populations than in seronegative controls. These data demonstrated that even when viral load is below the limit of detection, changes in immune cells may persist. The shift of immune cells to a more proinflammatory state could contribute to the comorbidities observed in HIV-1 individuals despite ART [6, 37]. It will be important to study individuals at variable times after therapy initiation to determine if and when immune cell states return to pre-infection levels.

Finally, we applied this technology to a primary CD4+ T cell model of early HIV-1 infection to identify genes that directly contribute to sensitivity or resistance to HIV-1 infection. We identified two long-noncoding RNAs, *NEAT1* and *MALAT1* that correlated with HIV-1 transcript levels. These two lncRNAs have previously been shown to regulate T cell activation and control HIV-1 transcription, but their role in regulating the cell sensitivity to HIV-1 infection has not been determined [28, 29, 38]. Other studies using single-cell genomic approaches have determined that HIV-1-infected cells undergo clonal expansion due to antigenic or cytokine stimulation that help with seeding the latent reservoir that are increased over time [39]. Understanding the clonal T cell dynamics during infection and during therapy will be important for future studies. Moreover, studies have identified that the proviruses that make up the latent reservoir are enriched in sequences that are present near ART initiation [40, 41]. This could suggest regulation of long-term persistence of HIV-infected cells. The genes identified in our primary model of acute infection could indicate that there are cellular pathways that restrict HIV replication and establishment of persisting infected cells for long-term. These genes, along with RNA splicing factors identified in our analysis, may be targeted for future therapies to block HIV-1 infection or control viral transcription in HIV-1 cure strategies.

There are limitations to our study, the major one is that small exploratory study size. We know that demographic and other host factors such as age, sex, race/ethnicity, other health conditions all can effect host immune cell profiles. Moreover, the comparison of ART drug regimens and individuals with different duration of ART would be informative that was not addressed here. Larger studies that control for infection history ART class and duration as well as account for the impact of demographics on immune cell profiles will be required to validate any discoveries. Transcripts that have lower expression in the single-cell datasets could also be critical for HIV immune regulation but could have the impact reduced due to low and variable detection. Improved scRNA-seq sensitivity or targeted approaches on selected low-abundant transcripts could highlight the significance of these genes. Another limitation is we studied the immune cells in only the peripheral blood. Determining how HIV-1 infection and treatment influences cells in other tissues such as the gut and lymphoid organs will be critical in understanding how the virus impacts immune cells in these critical tissue environments.

## Conclusions

In summary, our study demonstrates the ability of scRNA-seq to overcome the obstacles of the complexity and heterogeneity of the immune system. Using this approach, we generated a high-resolution atlas of the molecular changes that occur in blood immune cells during HIV-1 infection and ART treatment, uncovering novel genes and inflammatory pathways that may be important for restoring immune homeostasis in individuals with HIV-1 infection. Integration of the HIV-1 viral genes to identify infected cells and determine transcriptome changes that occur during infection of the target cells provides a framework to study HIV-1 and other viral infections such as influenza, SARS-CoV-2 and measles that are known to perturb the gene expression of host cells. Taken together, using this approach in other infection and disease contexts will reveal unique and overlapping immune pathways that could be targeted with drugs or treatment in the future.

## Methods

### Experimental model and subject details

Deidentified peripheral blood mononuclear cell (PBMC) samples were used from existing cohorts of HIV-1-infected and seronegative individuals stored at the Duke Human Vaccine Institute that were approved by the Duke Medicine Institutional Review Boards as well as the ethics boards of the local sites. Individuals were mostly male and of African descent (Table 1). These individuals were a part of a large HIV-1 clinical study that received longitudinal tissue sample collection. Whole venous blood was drawn and then fractionated into PBMCs and cryopreserved. This was an exploratory study, and the selected participants were not statistically matched for external factors such as age, sex, viral load or ethnicity. Future studies should be performed to confirm these cofounders. Cells from six untreated HIV-infected, three ART-treated HIV-1-infected (HIV-1 viral load below the limit of detection), and three HIV-1 seronegative (control) individuals were used to perform single-cell RNA sequencing studies (10× Genomics; Table 1).

### Primary cell model of HIV-1 infection

PBMCs from two different HIV-1 seronegative individuals (not included in other analysis) were thawed and T cells were activated by stimulation with CD3 (clone OKT3; eBioscience, 150 ng/mL) and CD28 (clone CD28.2; BD Biosciences, 150 ng/mL) antibodies in RPMI media supplemented with 20% FBS and recombinant human IL-2 (NIH AIDS Reagent Program, Division of AIDS, NIAID, National Institutes of Health, 30 U/mL). Activation was allowed to proceed for 72 h at 37 °C 5% CO_2_. CD4+ T cells were then enriched by removal of CD8+ T cells using magnetic CD8 microbeads (Miltenyi Biotec, Germany). Cells were then infected with full-length HIV-1 infectious molecular clone virus derived from an infected individual CH058 [26] by spinoculation at 1200×*g* for 2 h at room temperature [42]. Infected cells were incubated in RPMI media supplemented with 20% FBS and 30 U/mL IL-2 for 24 h. Cells were then harvested and washed with PBS for scRNA-seq.

### scRNA-seq library construction and sequencing

PBMCs were thawed, washed and placed in single-cell suspensions with PBS + 0.04% BSA. Single-cells after activation and 24 h infection from the primary cell model of HIV-1 infection were also washed and also placed in single-cell suspension with PBS + 0.04% BSA. Cellular suspensions were loaded on a GemCode Single-Cell instrument (10× Genomics) to generate single-cell beads in emulsion. Single-cell RNA-seq libraries were then prepared using a GemCode Single Cell 3’ Gel bead and library kit version 2 (10× Genomics). Single-cell barcoded cDNA libraries were quantified by quantitative PCR (Kappa Biosystems) and sequenced on an Illumina NextSeq 500 [9, 14, 43, 44]. Read lengths were 26 bp for read 1, 8 bp i7 index, and 98 bp read 2. Cells were sequenced to greater than 50,000 reads per cell.

### scRNA-seq mapping and analysis

After sequencing, the Cell Ranger Single Cell Software Suite (version 2.1.1) was used to generate sequencing fastq files and to perform sample de-multiplexing, barcode processing, reference alignment and single-cell 3’ gene counting [45]. Reads were aligned to the human genome (GRCH38) combined with the consensus sequence of HIV-1 clade C *gag* derived from viruses in the HIV Sequence Database (https://www.hiv.lanl.gov). Clade C was selected due to it being the predominant circulating HIV-1 strain in southern Africa where most of the individuals originate. Samples were aggregated using the CellRanger Aggr function to create a single matrix of cell barcodes and gene counts for the groups. During the process each library is normalized for mapped sequencing depth. Reads are subsampled from higher-depth libraries until they all have an equal number of reads per cell that are confidently mapped to the transcriptome in order to control for variation in the number of reads per sample (sequencing depth). Finally, to correct for any batch effects we used the Seurat analysis pipeline Multi CCA method to regress out cell–cell variation in gene expression in order to control for technical variation. The union of variable genes across all individual samples are utilized to renormalize the data.

Matrices of cell barcodes and gene counts generated by Cell Ranger were loaded into Seurat R package (v3.2.3) for graph-based cell clustering, dimensionality reduction and data visualization [46–48]. We filtered low quality cells that had lower than 200 expressed transcripts and percentage of mitochondrial genes expressed greater than 20% and for the primary cell model we reduced this threshold to greater than 10% mitochondrial genes. We included up to 45 PCA dimensions for the PBMCs and 48 PCA for the primary cell model for downstream graph-based clustering and UMAP visualization. All other parameters we followed the default Seurat recommendations. SingleR (v. 1.0.5) was utilized to assist with immune cell type identification [49]. Supervised identification of cell identity and states were performed using Azimuth [50]. Cells were annotated using the RunAzimuth, a Seurat object was provided as a query, function available on azimuth package in R using default settings. The Human-PBMC dataset was used as the reference dataset for Azimuth [48]. The reported cell type annotations are based on “celltype.I2” Azimuth output. Differentially expressed genes between cell clusters or groups were determined using Seurat by the Wilcoxon rank sum test. Genes that correlated with HIV-1 transcript expression were calculated by Pearson correlation and corrected for multiple comparisons using Bonferroni. Graphs and plots were generated using the Seurat and ggplot2 (v3.3.3) R packages and Graphpad Prism version 8.

GSEA v4.1.0 was used for gene set enrichment analysis. For the top 2000 variable genes, raw counts were linearly transformed (using ScaleData function available on Seurat Package) and averaged to generate expression dataset. Next, phenotype file was prepared based on group information for the samples. Hallmark gene sets (v7.2) and Human_HGNV_ID_MiSigDB (v7.2) chip hosted on the GSEA-MSigDB file servers were used as Gene sets and Chip annotation file respectively. Genes weren’t collapsed and “gene_set” parameter was used for permutation. While rest of the options were run with default settings.

## Supplementary Information

**Additional file 1. Table S1**: Individual sample scRNA-seq results. **Table S2** Cell cluster computational inference of cell type. **Table S6** GSEA enrichment of untreated HIV-1 compared to control. **Table S7** GSEA enrichment of ART-treated HIV-1 compared to control. **Fig. S1**. scRNA-seq individual cell QC data. **Fig. S2**. Determining cell types and genes in each cluster. **Fig. S3**. NK and T cell reclustering.

**Additional file 2. Table S3**: Genes upregulated in each cluster in scRNA-seq total PBMC.

**Additional file 3. Table S4**: Genes differentially expressed between HIV-1-infected compared to seronegative.

**Additional file 4. Table S5**: Genes differentially expressed between HIV-1-treated compared to seronegative.

**Additional file 5. Table S8**: Genes upregulated in each cluster in scRNA-seq T and NK cells.

**Additional file 6. Table S9**: Genes differentially expressed between HIV-1-infected compared to seronegative CD4+ T cells.

**Additional file 7. Table S10**: Genes differentially expressed between HIV-1-infected compared to seronegative CD8+ T cells.

**Additional file 8. Table S11**: Genes differentially expressed between HIV-1-treated compared to seronegative CD4+ T cells.

**Additional file 9. Table S12**: Genes differentially expressed between HIV-1-treated compared to seronegative CD8+ T cells.

**Additional file 10. Table S13**: Genes correlated or anti-correlated with HIV-1 gag transcript expression.

## Data Availability

The single-cell RNA seq unprocessed reads have been deposited in the NCBI SRA database under the BioProject ID: PRJNA681021.

## Code availability

Code and other processed file formats are available from corresponding authors upon reasonable request.
