# Supplementary material for: Single-cell analysis of immune cell transcriptome during HIV-1 infection and therapy
Source: BMC Immunol. 2022 Sep 29;23:48. doi: 10.1186/s12865-022-00523-2 (PMC9520965; doi:10.1186/s12865-022-00523-2)
Supplement: Supplementary file 1 — Additional file 1. Table S1: Individual sample scRNA-seq results. Table S2 Cell cluster computational inference of cell type. Table S6 GSEA enrichment of untreated HIV-1 compared to control. Table S7 GSEA enrichment of ART-treated HIV-1 compared to control. Fig. S1. scRNA-seq individual cell QC data. Fig. S2. Determining cell types and genes in each cluster. Fig. S3. NK and T cell reclustering. [file 12865_2022_523_MOESM1_ESM.pdf]

**Pollara et al. Supplemental material**

**Table S1. Individual sample scRNA-seq results.**
**Table S2. Cell cluster computational inference of cell type.**
**Table S3. Genes upregulated in each cluster in scRNA-seq total PBMC.**
**Table S4. Genes differentially expressed between HIV-1-infected compared to seronegative.**
**Table S5. Genes differentially expressed between HIV-1-treated compared to seronegative.**
**Table S6. GSEA enrichment of untreated HIV-1 compared to control.**
**Table S7. GSEA enrichment of ART-treated HIV-1 compared to control.**
**Table S8. Genes upregulated in each cluster in scRNA-seq T and NK cells.**
**Table S9. Genes differentially expressed between HIV-1-infected compared to seronegative CD4+ T cells.**
**Table S10. Genes differentially expressed between HIV-1-infected compared to seronegative CD8+ T cells.**
**Table S11. Genes differentially expressed between HIV-1-treated compared to seronegative CD4+ T cells.**
**Table S12. Genes differentially expressed between HIV-1-treated compared to seronegative CD8+ T cells.**
**Table S13. Genes correlated or anti-correlated with HIV-1 *gag* transcript expression.**

**Figure S1. scRNA-seq individual cell QC data.**
**Figure S2. Determining cell types and genes in each cluster.**
**Figure S3. NK and T cell reclustering.**

**Table S1. Individual sample scRNA-seq results**

| Sample ID | Sample Group | Number of cells | Median UMI | Median Genes |
|---|---|---|---|---|
| 0036 | HIV-1 seronegative | 9274 | 2345 | 839.5 |
| 1363 | HIV-1 seronegative | 5392 | 3107.5 | 1023 |
| 1628 | HIV-1 seronegative | 10300 | 3928.5 | 1157 |
| 0501 | HIV-1 untreated | 7424 | 2578 | 1012 |
| 0010 | HIV-1 untreated | 5329 | 2770 | 995 |
| 0468 | HIV-1 untreated | 5089 | 2575 | 912 |
| 0694 | HIV-1 untreated | 4163 | 2628 | 961 |
| 0782 | HIV-1 untreated | 6608 | 1725 | 705 |
| 0391 | HIV-1 untreated | 3929 | 3569 | 1284 |
| 0592 | HIV-1 ART treated | 7354 | 4146.5 | 1162 |
| 0010 | HIV-1 ART treated | 10889 | 1807 | 723 |
| 0694 | HIV-1 ART treated | 5484 | 2218 | 810 |

**Table S2. Cell cluster computational inference of cell type**

| seurat_clusters | DatabaseImmuneCellExpressionData | MonacoImmuneData |
|---|---|---|
| 0 | T cells, CD4+ | CD4+ T cells |
| 1 | T cells, CD8+ | CD4+ T cells |
| 2 | NK cells | T cells |
| 3 | NK cells | NK cells |
| 4 | NK cells | T cells |
| 5 | Monocytes | Monocytes |
| 6 | T cells, CD4+ | T cells |
| 7 | B cells | B cells |
| 8 | Monocytes | Monocytes |
| 9 | Monocytes | Monocytes |
| 10 | NK cells | T cells |
| 11 | B cells | B cells |
| 12 | NK cells | T cells |
| 13 | NA | NA |
| 14 | NK cells | T cells |
| 15 | Monocytes | Monocytes |
| 16 | Monocytes | Monocytes |
| 17 | Monocytes | Dendritic cells |
| 18 | B cells | B cells |
| 19 | NK cells | NK cells |
| 20 | NA | Dendritic cells |
| 21 | NK cells | T cells |
| 22 | NK cells | Progenitors |

**Table S6. GSEA enrichment of untreated HIV-1 compared to control**

| NAME | SIZE | ES | NES | NOM p-val | FDR q-val | FWER p-val | RANK AT MAX | LEADING EDGE |
|---|---|---|---|---|---|---|---|---|
| HALLMARK_G2M_CHECKPOINT | 83 | 0.52524215 | 1.9201477 | 0 | 0.001 | 0.002 | 734 | tags=73%, list=37%, signal=111% |
| HALLMARK_MITOTIC_SPINDLE | 41 | 0.55815804 | 1.8087457 | 0 | 0.007 | 0.014 | 628 | tags=71%, list=31%, signal=101% |
| HALLMARK_P53_PATHWAY | 41 | 0.5254313 | 1.7345463 | 0 | 0.01 | 0.048 | 397 | tags=44%, list=20%, signal=54% |
| HALLMARK_INTERFERON_ALPHA_RESPONSE | 26 | 0.5657851 | 1.7312152 | 0.001 | 0.012 | 0.048 | 640 | tags=73%, list=32%, signal=106% |
| HALLMARK_COMPLEMENT | 59 | 0.4972665 | 1.7226 | 0 | 0.01 | 0.055 | 500 | tags=56%, list=25%, signal=72% |
| HALLMARK_FATTY_ACID_METABOLISM | 23 | 0.5732651 | 1.6822027 | 0.009 | 0.016 | 0.094 | 326 | tags=43%, list=16%, signal=51% |
| HALLMARK_CHOLESTEROL_HOMEOSTASIS | 16 | 0.6104012 | 1.663567 | 0.004 | 0.017 | 0.116 | 482 | tags=63%, list=24%, signal=82% |
| HALLMARK_XENOBIOTIC_METABOLISM | 39 | 0.48944244 | 1.6102582 | 0.013 | 0.027 | 0.201 | 486 | tags=51%, list=24%, signal=66% |
| HALLMARK_E2F_TARGETS | 98 | 0.42562306 | 1.5730865 | 0 | 0.036 | 0.285 | 691 | tags=55%, list=35%, signal=80% |
| HALLMARK_INFLAMMATORY_RESPONSE | 79 | 0.42635623 | 1.5351864 | 0.004 | 0.049 | 0.393 | 537 | tags=44%, list=27%, signal=58% |

**Table S7. GSEA enrichment of ART-treated HIV-1 compared to control**

| NAME | SIZE | ES | NES | NOM p-val | FDR q-val | FWER p-val | RANK AT MAX | LEADING EDGE |
|---|---|---|---|---|---|---|---|---|
| HALLMARK_TNFA_SIGNALING_VIA_NFKB | 99 | 0.73709977 | 3.1880076 | 0 | 0 | 0 | 392 | tags=79%, list=20%, signal=93% |
| HALLMARK_INFLAMMATORY_RESPONSE | 79 | 0.55249864 | 2.2961788 | 0 | 0 | 0 | 570 | tags=71%, list=29%, signal=95% |
| HALLMARK_HYPOXIA | 43 | 0.5799632 | 2.118108 | 0 | 7.44E-04 | 0.001 | 320 | tags=44%, list=16%, signal=51% |
| HALLMARK_ESTROGEN_RESPONSE_LATE | 50 | 0.52476484 | 2.0182989 | 0 | 0.00168096 | 0.004 | 393 | tags=38%, list=20%, signal=46% |
| HALLMARK_UV_RESPONSE_UP | 33 | 0.5574774 | 1.9103618 | 0 | 0.00456023 | 0.012 | 320 | tags=48%, list=16%, signal=57% |
| HALLMARK_KRAS_SIGNALING_UP | 51 | 0.4829574 | 1.8472776 | 0.00488998 | 0.00779019 | 0.024 | 489 | tags=53%, list=24%, signal=68% |
| HALLMARK_APOPTOSIS | 48 | 0.4899276 | 1.836889 | 0.00224215 | 0.00693623 | 0.025 | 320 | tags=48%, list=16%, signal=56% |
| HALLMARK_INTERFERON_GAMMA_RESPONSE | 68 | 0.44720253 | 1.7929685 | 0 | 0.01059651 | 0.044 | 528 | tags=53%, list=26%, signal=69% |
| HALLMARK_P53_PATHWAY | 41 | 0.48205066 | 1.7498927 | 0.00722892 | 0.0135565 | 0.062 | 436 | tags=51%, list=22%, signal=64% |
| HALLMARK_COMPLEMENT | 59 | 0.44515666 | 1.7428768 | 0.00248139 | 0.0133448 | 0.068 | 493 | tags=49%, list=25%, signal=63% |
| HALLMARK_ESTROGEN_RESPONSE_EARLY | 31 | 0.5083145 | 1.7285014 | 0.00451467 | 0.01336376 | 0.075 | 381 | tags=45%, list=19%, signal=55% |
| HALLMARK_EPITHELIAL_MESENCHYMAL_TRANSITION | 52 | 0.44602725 | 1.7277389 | 0.00668151 | 0.01225011 | 0.075 | 320 | tags=31%, list=16%, signal=36% |
| HALLMARK_COAGULATION | 36 | 0.4568763 | 1.6258614 | 0.01146789 | 0.02801298 | 0.174 | 386 | tags=36%, list=19%, signal=44% |
| HALLMARK_MITOTIC_SPINDLE | 41 | 0.44885015 | 1.6220896 | 0.00692841 | 0.02653278 | 0.176 | 171 | tags=17%, list=9%, signal=18% |
| HALLMARK_XENOBIOTIC_METABOLISM | 39 | 0.43573573 | 1.5763764 | 0.02505695 | 0.03518377 | 0.239 | 386 | tags=38%, list=19%, signal=47% |

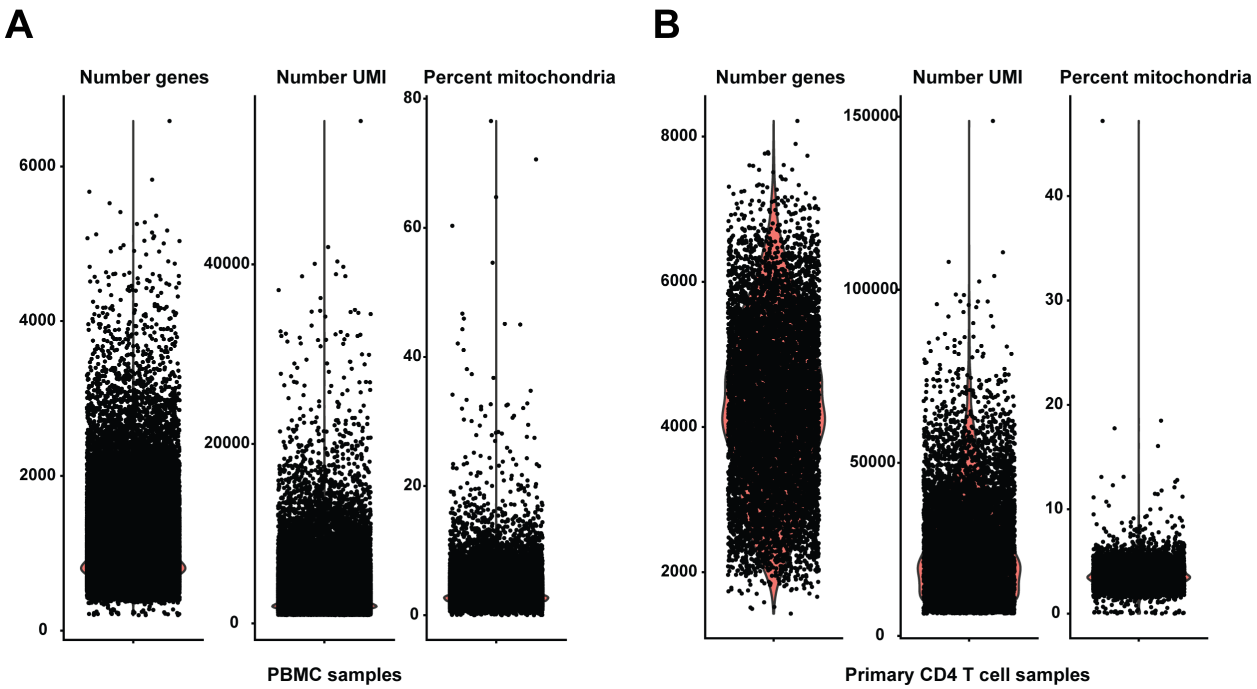


**Figure S1. scRNA-seq individual cell QC data. (A-B)** Plots of each cell in the **(A)** PBMC samples or **(B)** the primary CD4 T cell model samples for the number of genes detected, number of unique molecular identifiers (UMI) for each RNA molecule and the percentage of genes that are of mitochondrial origin. Each point represents an individual cell.

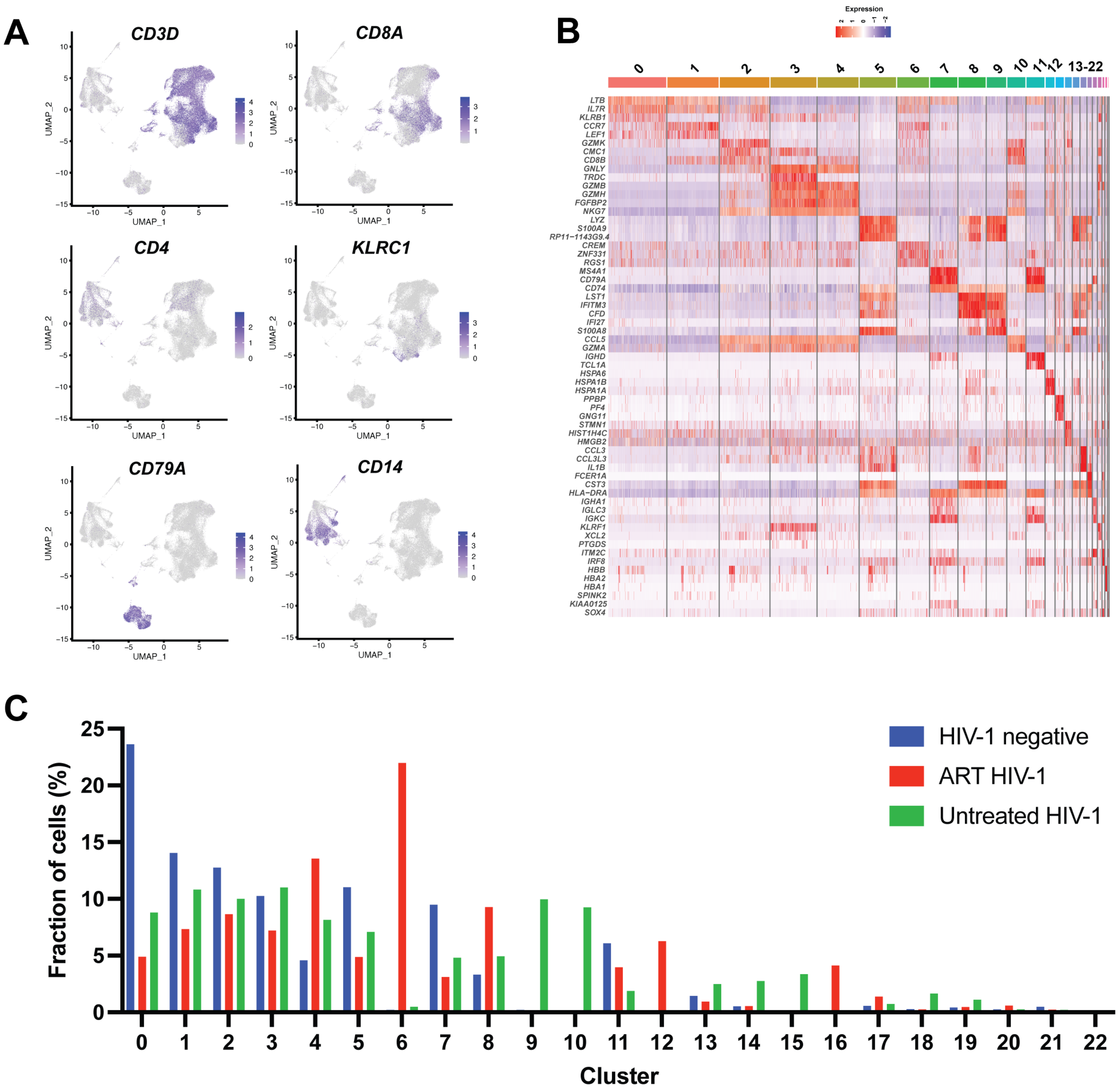


**Figure S2. Determining cell types and genes in each cluster. (A)** Expression of immune cell transcripts that are associated with cell identity used to identify clusters of cells. **(B)** Heatmap of the top 3 upregulated transcripts in each identified cluster. **(C)** Bar graph showing the fraction of total single cells in each transcriptional cluster by group.

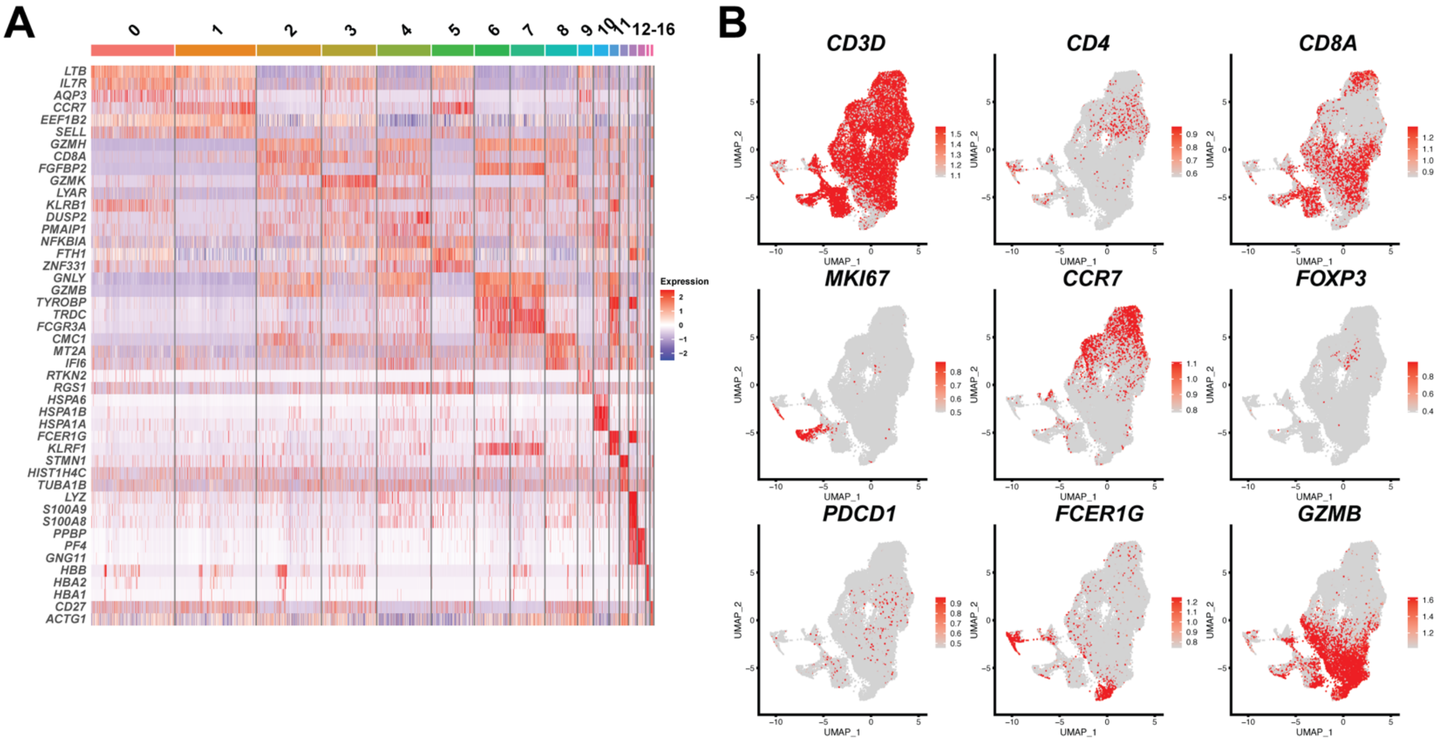


**Figure S3. NK and T cell reclustering. (A)** Heatmap of top 3 upregulated genes in each of the 17 clusters identified. **(B)** Heatmap of the top 5 upregulated transcripts in each identified cluster. **(C)** Expression of immune cell transcripts that are associated with cell identity used to identify clusters of cells.
